# H-NOX proteins in the virulence of pathogenic bacteria

**DOI:** 10.1042/BSR20212014

**Published:** 2022-01-06

**Authors:** Cameron Lee-Lopez, Erik Yukl

**Affiliations:** Department of Chemistry and Biochemistry, New Mexico State University, Las Cruces, NM 88003, U.S.A.

**Keywords:** Biofilm, heme, nitric oxide signalling

## Abstract

Nitric oxide (NO) is a toxic gas encountered by bacteria as a product of their own metabolism or as a result of a host immune response. Non-toxic concentrations of NO have been shown to initiate changes in bacterial behaviors such as the transition between planktonic and biofilm-associated lifestyles. The heme nitric oxide/oxygen binding proteins (H-NOX) are a widespread family of bacterial heme-based NO sensors that regulate biofilm formation in response to NO. The presence of H-NOX in several human pathogens combined with the importance of planktonic–biofilm transitions to virulence suggests that H-NOX sensing may be an important virulence factor in these organisms. Here we review the recent data on H-NOX NO signaling pathways with an emphasis on H-NOX homologs from pathogens and commensal organisms. The current state of the field is somewhat ambiguous regarding the role of H-NOX in pathogenesis. However, it is clear that H-NOX regulates biofilm in response to environmental factors and may promote persistence in the environments that serve as reservoirs for these pathogens. Finally, the evidence that large subgroups of H-NOX proteins may sense environmental signals besides NO is discussed within the context of a phylogenetic analysis of this large and diverse family.

## Introduction

Oxidative and nitrosative stress are encountered by virtually every organism, and mechanisms to sense and ameliorate these stresses are essential to survival. Nitric oxide (NO), in addition to being a cytotoxic agent of nitrosative stress, is an important signaling molecule at low (nM) concentration in both eukaryotes and prokaryotes. Two hemoproteins, heme nitric oxide/oxygen binding protein (H-NOX) and NosP, have been recently identified as important bacterial sensors for the mediation of communal responses, such as biofilm formation, in response to low concentrations of NO (see recent reviews [1–3]). Both hemoproteins appear to regulate metabolism of c-di-GMP, a uniquely bacterial secondary messenger that regulates processes such as motility, biofilm formation and virulence [4]. This molecule is synthesized from 2GTP by diguanylate cyclases (DGCs) and hydrolyzed by phosphodiesterases (PDEs). In some cases, H-NOX proteins directly influence the activity of these enzymes, while in others they bind and regulate histidine kinases (HKs) that modulate DGC/PDE activity through phosphorylation. The HK may also phosphorylate response regulators (RRs) that influence the transcription of target genes.

NO is utilized by the innate immune system as a defense against invading pathogens [5], and biofilms are associated with chronic infections and antibiotic resistance [6]. This combined with the presence of H-NOX proteins in several human pathogens suggests that they may be important virulence factors and potential targets for the development of novel antimicrobials. However, evidence of direct involvement of H-NOX with virulence is scarce and largely indirect through their association with biofilms. Here we summarize the relationship between biofilm formation and virulence and the current knowledge of how H-NOX signaling pathways may participate in this process. We go on to explore the possible roles of H-NOX proteins in the pathogenesis of *Vibrio cholerae, Vibrio parahaemolyticus* and *Legionella pneumophila*, and in the commensal colonization of squid by *Vibrio fischeri*. The diversity of H-NOX sequences among bacteria and possible alternative signals are then presented. Finally, the future of these proteins as antimicrobial targets is discussed.

## Biofilm formation and virulence

A biofilm is an aggregate of bacteria within a matrix of extracellular polymeric substance (EPS) [7]. This EPS provides a protective barrier that can enable bacterial persistence in various environments including those of clinical relevance such as potable water distribution systems [8] and on medical devices and implants [9]. Cells within biofilm also exhibit enhanced resistance to antibiotics due to their limited transport through the biofilm [10] and enhanced efflux [11]. As biofilms reach maturity, daughter cells shed off the main mass as planktonic cells, providing a means of transmission to new environments and hosts. Thus, antibiofilm strategies may become an important weapon in the fight against antibiotic resistance and chronic bacterial infections.

The transition between planktonic and biofilm-associated lifestyles is highly regulated by a complex suite of factors. NO has emerged as an important environmental signal regulating biofilm formation. Indeed, treatment with NO can induce the dispersal of *Pseudomonas aeruginosa* biofilms [12,13] and sensitize biofilms of other pathogenic species to chlorine treatment [14]. These promising indications highlight the importance of understanding NO signaling and biofilm regulation in pathogens, processes mediated at least in part by H-NOX proteins as discussed below.

## H-NOX functions and signaling pathways

The bacterial H-NOX proteins were originally identified by homology with the heme domain of soluble guanylate cyclase (sGC) [15], a eukaryotic enzyme that synthesizes the secondary messenger cyclic GMP (cGMP) in response to NO [16]. Like sGC, the H-NOX proteins from facultative anaerobes exclude oxygen and bind NO specifically, usually as 5-coordinate ferrous–nitrosyl complexes. In contrast, H-NOX proteins from obligate anaerobes bind to O_2_, forming stable, 6-coordinate ferrous–oxy complexes [17–19]. The latter group is characterized by the presence of a Tyr residue in the distal pocket that is requisite for O_2_ binding [20–22]. These proteins are membrane bound and fused to methyl-accepting chemotaxis domains, and it is likely that they act as O_2_ sensors for chemotaxis signaling [23]. Conformational changes associated with NO or O_2_ binding have been determined for several H-NOXs by X-ray crystallography and have been reviewed in detail elsewhere [24]. Briefly, the activation mechanism proceeds through displacement and rotation of distal and proximal subdomains about a hinge composed of two conserved Gly residues. No high-resolution structures exist of an H-NOX protein in complex with its signaling partner. However, hydrogen-deuterium exchange mass spectrometry (HDX-MS) [25,26] and NMR experiments [27] indicate that H-NOX interacts through both subdomains, consistent with their re-orientation modulating target activity.

**Figure 1:**
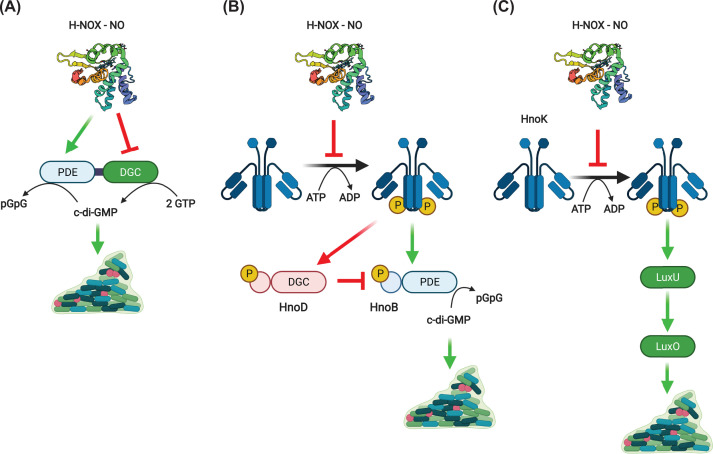
H-NOX signaling pathways H-NOX proteins may directly influence the activities of (**A**) DGC and/or PDE proteins or (**B**) HKs to alter c-di-GMP levels and biofilm formation. (**C**) H-NOX/HK signaling pathways may also participate directly in quorum sensing pathways. Created with BioRender.com.

Here we will confine our discussion to the NO-binding H-NOX proteins from facultative anaerobes. These appear to influence biofilm formation through signaling pathways that alter the intracellular concentration of c-di-GMP. These *hnox* genes are usually adjacent to those of HKs or DGC/PDE enzymes. Three general pathways can be outlined in which most H-NOX proteins studied to date appear to participate (Figure 1 and Table 1). H-NOX can interact directly with DGC/PDE enzymes, inhibiting the DGC functionality and/or stimulating PDE activity resulting in a decrease in c-di-GMP concentrations and a dissolution of biofilm in response to NO (Figure 1A). This pathway is active in *Legionella pneumophila* [28] and *Shewanella woodyi* [29], although in the former organism the PDE domain is likely inactive. Another variation of this pathway is observed in *Paracoccus denitrificans* [30], where c-di-GMP appears to negatively regulate biofilm formation.

**Table 1:** Characterized H-NOX proteins from facultative anaerobes and their signaling partners

Organism	H-NOX Uniprot ID [41]	Signaling partner	Knockout phenotype
*Legionella pneumophila* [28]	LPG2459	HK (LPG2458)	Comparable with WT
*Legionella pneumophila* [28]	LPG1056	DGC/PDE (LPG1057)	Hyperbiofilm
*Paraccocus denitrificans* [30]	PDEN_3719	DGC (PDEN_3720)	Impaired biofilm formation
*Pseudoalteromonas atlantica* [33]	PATL1532	HK (PATL1533)	Unknown effect on c-di-GMP pools through response regulator (PATL1534)
*Saccharophagus degradans* [32]	SDE_3804	HK (SDE_3803)	Unknown
*Saccharophagus degradans* [32]	SDE_3557	HK (SDE_3803)	Unknown
*Shewanella oneidensis* [31]	SO2144	HK (SO2145)	Reduced biofilm formation in response to NO
*Shewanella woodyi* [29]	SWOO_2751	DGC/PDE (SWOO_2750)	Impaired biofilm formation
*Silicibacter sp.* [34]	not found	HK	Unknown
*Vibrio cholerae* [31]	VCA0720	HK (VCA0719)	n/d
*Vibrio fischeri* [39]	VF_A0071	HK (VF_A0072)	Enhanced growth and host colonization
*Vibrio harveyi* [36]	VIBHAR_01911	HK (VIBHAR_01913)	Impaired quorum sensing indicated by a lack of bioluminescence
*Vibrio parahaemolyticus* [37]	VP1877	HK (VP1876)	Unknown

n/d: unknown (not determined).

The second pathway relies on a phosphorelay originating with the H-NOX adjacent HK and resulting in activation of PDE activity and a decrease in c-di-GMP (Figure 1B). The HK also phosphorylates and deactivates an allosteric PDE inhibitor HnoD, which itself has an inactive DGC domain. NO-bound H-NOX inhibits HK autophosphorylation and subsequent pathway steps, resulting in an increase in c-di-GMP and biofilm formation in the presence of NO. This pathway has been demonstrated in *V. cholerae* and *Shewanella oneidensis* [31] and is likely active in *Saccharophagus degradans* [32] and *Pseudoalteromonas atlantica* [33] based on homology. The target of a similar pathway in *Silicibacter* sp. is an active DGC that is inhibited by phosphorylation [34]. A related pathway may be associated with a second H-NOX in *L. pneumophila*, although a null mutant exhibited no clear phenotype and further characterization was not pursued [28]. In *S. oneidensis*, a transcription factor HnoC is also phosphorylated, relieving its repression of the *hno* regulon and forming a transcriptional feedback loop [35]. The *hnoC* gene is absent from the *V. cholerae* genome.

**Figure 2:**
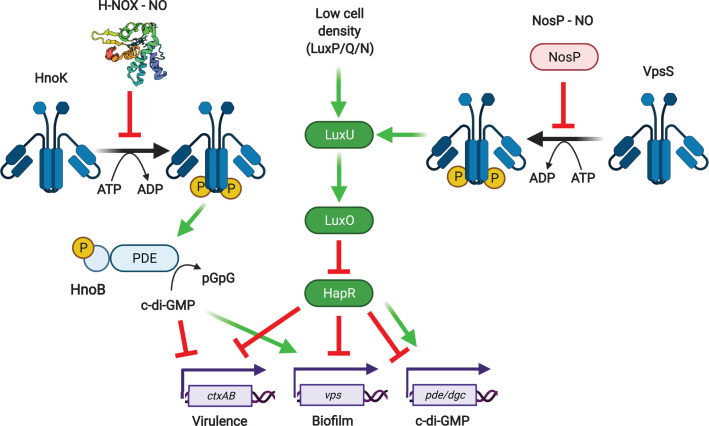
Intersecting signaling pathways in *V. cholerae* Green arrows indicate activating phosphorylation or gene induction events, while red bars indicate inhibition of various processes. Created with BioRender.com

A third pathway involves direct participation in quorum sensing pathways (Figure 1C). In *Vibrio harveyi* [36] and *Vibrio parahemolyticus* [37], the H-NOX regulated HK phosphorylates LuxU, a kinase involved in quorum sensing and bioluminescence. This circuit was further shown in *V. harveyi* to influence biofilm formation and modulate the expression of genes encoding flagellar motor proteins. Quorum sensing pathways also intersect the H-NOX pathway in *V. cholerae* through the action of a second heme-based NO sensor NosP [38] (Figure 2). Finally, H-NOX/HK signaling in *Vibrio fischeri* appears to regulate expression of genes encoding iron uptake and utilization proteins [39]. The details of this pathway were not elucidated in this case, but H-NOX signaling was demonstrated to alter this organism’s ability to colonize its squid host. In addition, H-NOX signaling inhibits biofilm formation in *V. fischeri* [40]. In this case, the H-NOX regulated HK positively regulates expression of the *syp* locus involved in biofilm formation. This is discussed in more detail below (Figure 4).

## H-NOX and virulence in *V. cholerae*

*V. cholerae* is the causative agent of the diarrheal disease cholera, which globally affects several million people and often results in more than 100000 deaths annually [42]. Infection usually occurs through the ingestion of contaminated drinking water [43] and can lead to sweeping epidemics, particularly in areas with poor sanitation and in tropical regions where the organism is endemic [44,45]. Biofilm formation is an important mechanism of *V. cholerae* survival in the aquatic environment and is known to enhance transmission and infectivity (reviewed in [46]). Further, it is established that NO is produced by the human host during *V. cholerae* infection [47]. Given the likely role of H-NOX in NO sensing and biofilm formation, it seems logical that the H-NOX signaling pathway may contribute to the pathogenesis of this organism.

However, this statement is made with some caution. H-NOX and NosP NO signaling, quorum sensing, biofilm formation, c-di-GMP metabolism and virulence factor expression are all part of an exceedingly complex network of signaling pathways, in which quorum sensing appears to play a central role (Figure 2). At low cell densities, quorum sensors act as kinases, activating a pathway that results in the expression of quorum regulatory RNAs (Qrrs) that destabilize the mRNA for the master transcription regulator HapR [48]. HapR inhibits expression of biofilm and virulence genes, including the cholera toxin genes *ctxAB* [49]. Consequently, low cell density favors biofilm formation and virulence gene expression through quorum sensing. At high cell density, the flow of phosphorylation is reversed, HapR is expressed, and biofilm and virulence genes are repressed. Quorum sensing also modulates the expression of 14 genes involved in c-di-GMP metabolism (DGC and PDE genes), including HnoB (VC1086) whose expression is directly activated by HapR [50]. Although no clear pattern of DGC vs. PDE expression was evident from this study, it was shown that a *luxO* mutation D47E mimicking low cell density exhibited a substantial increase in intracellular c-di-GMP relative to WT. This was also observed in a *ΔhapR* strain [51], suggesting that quorum sensing in the low cell density state also favors biofilm formation by HapR modulation of PDE/DGC expression resulting in increased c-di-GMP [52]. High c-di-GMP was shown to inhibit expression of virulence factors [53], in contrast with the effect of low cell density quorum signaling on HapR-mediated repression.

NosP intersects with this pathway by inhibiting the HK VpsS when bound to NO. VpsS contributes to low cell density quorum sensing and its overexpression leads to enhanced biofilm formation [54]. Consequently, NosP-dependent NO signaling would be expected to favor biofilm dispersal and virulence factor expression. Indeed, nanomolar concentrations of the NO donor sodium nitroprusside (SNP) were demonstrated to be effective in dispersing *V. cholerae* biofilms [14], although the role of NosP in this process has not been directly investigated. On the other hand, H-NOX NO signaling is predicted to inhibit HnoB (VC1086) PDE activity [31], presumably promoting biofilm formation as has been shown in the homologous system in *S. oneidensis.* However, it is worth noting that the *V. cholerae* genome encodes over 50 PDE and DGC-domain containing proteins [55], and the influence of H-NOX inhibition of a single PDE on biofilm formation in this organism has also not been directly evaluated. In any case, it is likely that both heme-based NO sensors participate in a highly complex signaling system that allows *V. cholerae* to adapt to the changing conditions of its pathogenic lifecycle.

It may be that each sensor plays distinct roles in response to host-derived or endogenous NO at different times in this cycle. Production of c-di-GMP late in the infection cycle has also been implicated in surviving the transition from host to environment [56]. Thus, H-NOX signaling may have a greater role in environmental adaptation and survival than in virulence *per se*. There is also some *in vitro* evidence that *V. cholerae* H-NOX may mediate signaling in response to oxidative stress as well through a heme-independent mechanism involving reversible disulfide bond formation [57,58]. To this end, it is interesting to note that a recent study identified the catalase gene *katB* as part of the c-di-GMP regulon [59]. High levels of the secondary messenger led to increased catalase expression and activity along with increasing survival after challenge with H_2_O_2_. However, in this study, elevated c-di-GMP was driven by expression of a heterologous DGC, and direct treatment of *V. cholerae* with H_2_O_2_ did not result in a significant increase in c-di-GMP.

## H-NOX and virulence in *Vibrio parahaemolyticus*

**Figure 3:**
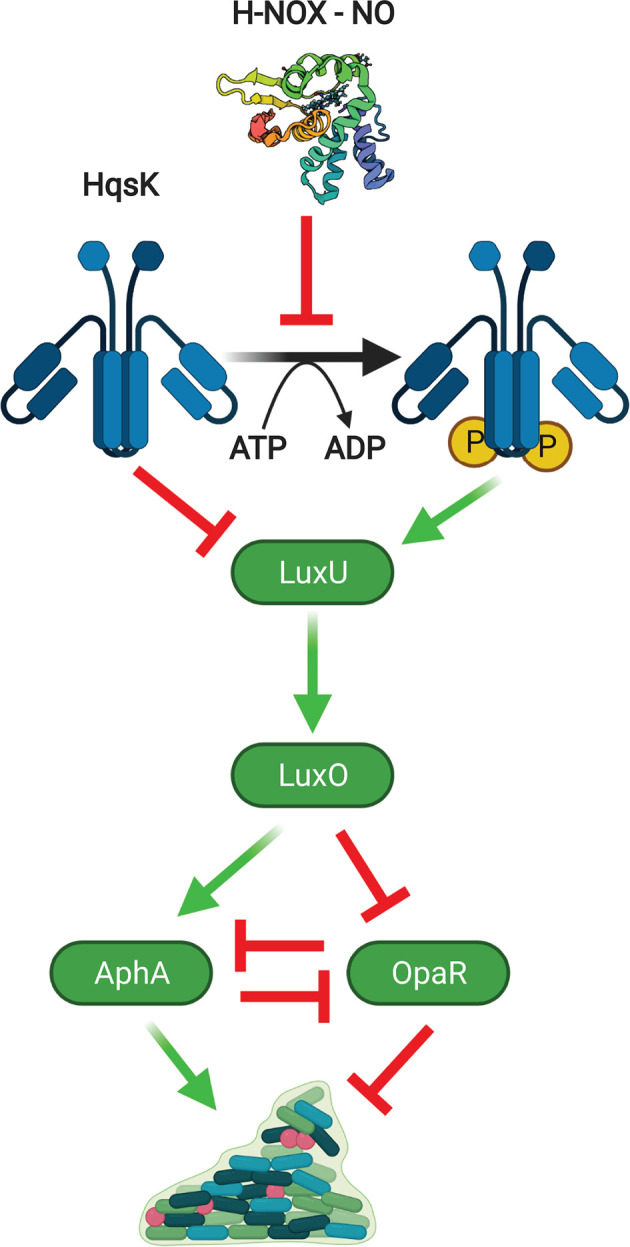
H-NOX signaling pathway in *V. parahaemolyticus* Green arrows indicate activating phosphorylation or gene induction events, while red bars indicate inhibition of various processes. Created with BioRender.com

*Vibrio parahaemolyticus* is a marine organism that was first identified in a case of food poisoning in Japan in 1950. Since then, it has been noted as one of the leading causes of foodborne illnesses [60]. *V. parahaemolyticus* H-NOX regulates the quorum sensing circuit (Figure 3) in a manner analogous to that of NosP in *V. cholerae* (Figure 2) through interaction with the hybrid kinase/phosphatase HqsK [37]. In the absence of NO, HqsK acts as a kinase to phosphorylate LuxU, participating in a phosphotransfer pathway also initiated by low cell density. As in *V. cholerae*, this pathway leads to the transcription of Qrrs, which bind and stabilize the mRNA of transcription factor *aphA* while destabilizing that of *opaR*, the *hapR* homolog in *V. parahaemolyticus*. Thus, OpaR acts as the master regulator at high cell density while AphA is predominant at low cell density. These transcription factors reciprocally inhibit each other’s transcription and thus comprise a master switch for gene expression between low and high cell density states.

Among numerous other functions [61], OpaR represses biofilm formation at high cell densities [62] although this property may be strain dependent. AphA is the predominant regulator at low cell densities, and in *V. cholerae* it positively regulates genes involved in biofilm formation [63] and virulence [64]. Likewise, AphA in *V. parahaemolyticus* is required for biofilm formation and effective colonization of a mouse infection model [65,66]. Since NO-bound H-NOX inhibits HqsK phosphorylation of LuxU, NO would seem to act as an autoinducer mimicking high cell density, favoring the OpaR regulon, decreased biofilm formation and virulence. Indeed, *opaR* transcription was up-regulated in response to NO, but no corresponding decrease in *aphA* was observed [37]. To our knowledge, no data have yet emerged on the response of *V. parahaemolyticus* biofilms to NO nor on the phenotype of an *hnox* deletion. Thus, the importance of H-NOX signaling to biofilm formation and virulence in this organism remains to be elucidated.

## H-NOX and host colonization by *V. fischeri*

**Figure 4:**
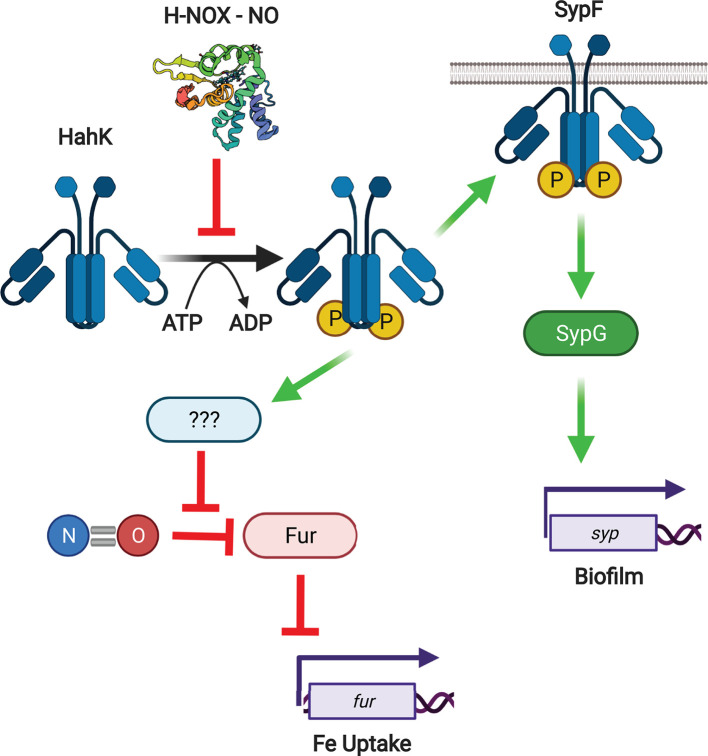
NO signaling pathways in *V. fischeri* Green arrows indicate activating phosphorylation or gene induction events, while red bars indicate inhibition of various processes. The mechanism by which HahK signaling represses the *fur* regulon is undetermined as indicated by question marks. Created with BioRender.com

Although not pathogenic, the H-NOX signaling system in *V. fischeri* (Figure 4) is worth exploring for several reasons. First, it colonizes a squid host (*Euprymna scolopes*) in a commensal relationship with many parallels to pathogenic infection. Secondly, the signaling output appears to be unusual relative to other H-NOX proteins that have been more extensively studied. Namely, H-NOX seems to suppress expression of genes of the ferric uptake regulator (Fur) regulon involved in iron uptake [39]. The signaling pathway responsible for this effect was not elucidated but may involve countering the destructive effect of Fur nitrosylation that is observed in *Escherichia coli* [67,68]. Finally, a *hnox* disrupted strain was actually more effective in colonizing the squid host than WT, an effect that was reversed in the presence of excess hemin and iron. This is the only example to our knowledge of experimental evidence demonstrating a role for H-NOX in influencing fitness for colonization of a host. The authors suggest that H-NOX functions to suppress iron uptake genes during initial colonization of the squid, thereby protecting themselves from damaging Fenton chemistry resulting from iron accumulation and host-derived hydrogen peroxide. Here again, it is interesting to note the intersection between H-NOX signaling and oxidative stress.

Biofilm formation is also important during the initial steps in colonization [69,70]. HahK, the HK adjacent to the *hnoX* gene in *V. fischeri*, was recently shown to be a positive regulator of biofilm formation. This likely occurs through phosphorylation of the histidine phosphotransferase domain of SypF and subsequent induction of the *syp* regulon, which encodes proteins involved in synthesis of exopolysaccharides important for biofilm formation [40]. H-NOX inhibited this pathway in an NO-dependent manner, and biofilm formation both in culture and in the host were also inhibited. Thus, the improved colonization efficiency of the *hnoX* mutant is likely due, at least in part, to enhanced biofilm formation. Although *V. fischeri* is non-pathogenic, it is easy to see how similar H-NOX signaling pathways might be relevant to virulence in other organisms. It remains to be seen whether these pathways are operative in any human pathogens.

## H-NOX and virulence in *L. pneumophila*

*L. pneumophila* is the causative of Legionnaires’ disease, a severe form of pneumonia often acquired by inhalation of contaminated freshwater aerosols [71]. *L. pneumophila* persistence in freshwater environments is aided by intracellular infection of amoebae [72] and association with multispecies biofilms [73]. Both environments could lead to exposure to low concentrations of NO.

**Figure 5:**
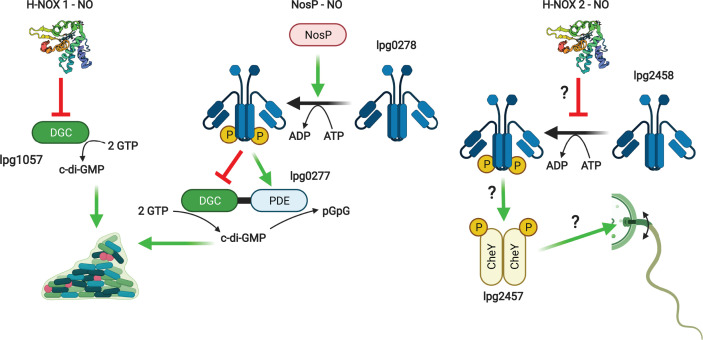
NO signaling pathways in *L. pneumophila* Green arrows indicate activating phosphorylation or binding events, while red bars indicate inhibition of various processes. The H-NOX 2 signaling pathway at right is purely hypothetical as indicated by question marks. Created with BioRender.com

The genome of *L. pneumophila* encodes two *hnox* (lpg1056 and lpg2459) and one *nosP* (lpg0270) gene (Table 1) that likely participate in NO signaling (Figure 5). Only one H-NOX (lpg1056), hereafter referred to as H-NOX 1, has been implicated in NO sensing and modulation of biofilm formation [28]. Specifically, NO-bound H-NOX inhibits DGC activity of lpg1057 and its deletion results in a hyperbiofilm phenotype but had no effect on growth in media nor in mouse macrophages or amoeba hosts. Non-toxic concentrations of NO were observed to have a modest inhibitory effect on biofilm formation in the *L. pneumophila* Lens strain that was dramatically enhanced in a *Δlpl1054* (equivalent to lpg1057) mutant, suggesting that this DGC is important for biofilm maintenance in the presence of NO [74]. Presumably, H-NOX represses this function in the presence of NO. Intriguingly, this study also revealed that some PDEs were associated with biofilm formation and that global c-di-GMP levels did not necessarily correlate with biofilm formation. This lends support to the view that local spatiotemporal fluctuations in c-di-GMP pools are important in signaling and may not be easily observed by interrogating the global c-di-GMP pool [75–77].

Similarly, NosP-NO stimulates a HK (lpg0278) that phosphorylates a bifunctional DGC/PDE lpg0277, inhibiting DGC activity and stimulating PDE activity [78]. The corresponding *Δlpl0329* deletion in the Lens strain exhibited increased biofilm formation [74], suggesting a predominant function in biofilm repression that would presumably be enhanced by NosP in the presence of NO. The response of this strain to NO was not evaluated, nor to our knowledge was a *ΔnosP* strain.

Finally, the function of H-NOX 2 remains ambiguous as a *Δhnox2* deletion strain had no effect on biofilm formation. The gene is adjacent to an HK (lpg2458) and CheY-like response regulator (lpg2457) suggesting a signaling pathway (Figure 5) that has not been experimentally confirmed. Some RRs are linked to DNA-binding domains and can influence transcription [79]. However, lpg2457 encodes only the CheY-like domain, and its unpublished crystal structure (PDB ID: 2QVG) is very similar to that of CheY from *E. coli* [80], which is known to bind flagellar motor proteins upon phosphorylation to influence chemotaxis [81]. Intriguingly, *L. pneumophila* lacks the sensor *cheA* as well as other mediators of chemotaxis [82], making it tempting to speculate that H-NOX 2 may act in this capacity, modifying CheY phosphorylation and chemotaxis in response to NO. However, there is no experimental evidence to date to support this.

## Alternative signals and sensing mechanisms for H-NOX

**Figure 6:**
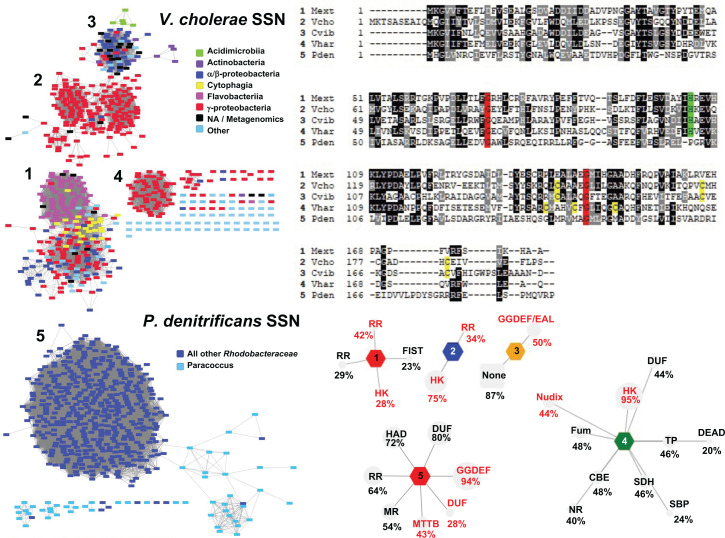
H-NOX sequence relationships and genome neighborhoods (Left) SSNs 1–5 were filtered to include only high confidence edges (E-values < 10^−47^). Sequences are represented by rectangles colored according to class, phyla or genus as indicated. (Top right) Also shown are sequence alignments from representatives of each cluster with potential zinc ligating Cys residues highlighted in yellow, the heme-binding His in green and Gly hinges in red. Species name abbreviations are Mext, *Methylobacterium extorquens*; Vcho, *Vibrio cholerae*; Cvib, *Caulobacter vibrioides*; Vhar, *Vibrio harveyi*; Pden, *Paracoccus denitrificans*. (Bottom right) A genome neighborhood network was constructed for each group where the gray spoke nodes indicate the prevalence of Pfam [83] protein family genes within five genes of *hnox* in at least 20% of genomes. The actual percentage of genomes with this genetic organization are indicated next to Pfam families. Those indicated in red have a median distance of one gene from the H-NOX, indicating that they are usually adjacent [57]. Abbreviations used for Pfam families are: CBE, chorismite-binding enzyme; DEAD, DEAD box helicase; DUF, domain of unknown function; GGDEF, diguanylate cyclase; EAL, phosphodiesterase; FIST, F-box and intracellular signal transduction; Fum, fumarase; HAD, haloacid dehydrogenase; MR, heavy metal resistance; MTTB, trimethylamine methyltransferase; NR, nitroreductase; Nudix, NUcleoside DIphosphate linked to X; SBP, solute-binding protein; SDH, serine dehydratase; TP, Trp/Tyr permease. Figure adapted from [57].

We have alluded to the possibility that some H-NOX proteins may mediate a response to signals other than, or in addition to, gaseous NO. *In vitro*, a heme-free form of *V. cholerae* H-NOX can reversibly form disulfide bonds between Cys residues at a zinc-binding site in response to oxidants [57,58]. Oxidation results in inhibition of the HK at a level comparable with the Fe(II)-NO state. In our recent work, we constructed a sequence similarity network (SSN) (Figure 6) from *V. cholerae* H-NOX, to identify four clusters of H-NOX proteins among several bacterial taxa [57]. The heme-binding proximal His was absolutely conserved, suggesting that these homologs bind heme and can function to sense NO. Several Gram-positive *Actinobacteria* from the *Acidimicrobiaceae, Nocardioidaceae, Micrococcaceae* and *Mycobacteriaceae* families were identified in group 3. Most of these are not characterized to the species level, making it difficult to ascertain whether H-NOX might be involved in biofilm regulation or pathogenesis. To our knowledge, there are no reports of putative NO-sensing H-NOX proteins from Gram-positive species, making this a potentially interesting area of inquiry. Finally, the presence of conserved 3Cys motifs in groups 2–4 suggest zinc-binding sites that could act as redox sensors. It will be interesting to determine if these proteins may confer resistance to oxidative stress encountered in the host organisms, although differentiating heme-free and heme-independent mechanisms *in vivo* is likely to prove extremely challenging.

Recently, an H-NOX signaling pathway was described in the non-pathogenic organism *Paracoccus denitrificans* [30]. The pathway is likely similar to that shown in Figure 1A, where NO-bound H-NOX inhibits a DGC. However, in this organism c-di-GMP appears to repress biofilm formation as the *ΔhnoX* mutant exhibits a biofilm-deficient phenotype whereas the *Δdgc* mutant exhibits a hyperbiofilm phenotype. Using the sequence of *P. denitrificans* H-NOX to construct a new SSN reveals a fifth, very large cluster of H-NOX sequences (Group 5, Figure 6). Remarkably, fewer than 10% of group 5 sequences have a proximal His residue, most being substituted with Pro. These sequences also lack any conserved Cys residues, which begs the question of how these proteins sense NO or any other environmental signal. Intriguingly, the Gly residues proposed to act as hinges between subdomains are conserved in all groups as are several other Gly, Pro and bulky hydrophobic residues, suggesting H-NOX proteins share a similar fold and mechanism of activation, although the triggers for activation may differ.

As noted previously, most H-NOX proteins studied to date influence behavior in response to NO by interaction with HK/RR systems and/or DGC/PDE enzymes (the latter contain GGDEF/EAL domains, respectively). This is represented in the genome network neighborhoods (GNN) for each group that show the frequency with which certain gene families are found in proximity to *hnox*. While there appear to be preferences for one pathway over another between different groups, it should be noted that these are not mutually exclusive. Also identified in group 1 are F-box and intracellular signal transduction (FIST) proteins, of which NosP is a member. NUcleoside DIphosphate linked to X (Nudix) proteins were identified in group 4. These hydrolases act on a wide variety of nucleoside diphosphate species [84]. To our knowledge, none are known to act on c-di-GMP, but their association with the H-NOX family and broad substrate specificity suggest the tantalizing possibility that they may be involved in c-di-GMP metabolism in these organisms.

Even among the relatively small number of organisms where H-NOX signaling has been studied in detail, surprising diversity exists. NO-bound H-NOX may lead to increases or decreases of c-di-GMP, which may promote or repress biofilm formation. They may do this through direct interaction with PDE/DGC enzymes, through phosphorelay cascades that regulate these enzymes, or through pathways that regulate gene expression. Given the large number of H-NOX sequences and potential signaling partners in bacteria, there is no doubt that further study will prove these proteins to be even more versatile than has been observed to date.

## H-NOX signaling in antimicrobial strategies

Figure 6 shows the impressive diversity and distribution of the H-NOX family across bacterial species. However, it appears that only a few known human pathogens encode H-NOX proteins. The fact that they mediate bacterial responses to NO and are implicated in biofilm formation makes it reasonable to expect that they may be important for virulence as well. To our knowledge, there is no direct evidence to suggest that this is the case. However, that biofilm formation can enhance resistance and survival in various environments indicates that this process may still be important for transmission from environmental reservoirs. Thus, exploiting NO-dependent biofilm regulation through H-NOX may lead to better water treatment strategies, as indicated by successful co-treatment of biofilms with NO and chlorination [14].

Both *V. cholerae* and *L. pneumophila* have complex life cycles involving distinct sessile and motile stages influencing pathogenesis. Establishing a model system for systematic study of virulence factors for either is notoriously difficult. This is particularly true of *V. cholerae*, which does not infect other mammals. Human intestinal organoids have recently shown promise as a tractable, humane model for *V. cholerae* infection [85] as well as other enteric bacterial diseases [86]. Breakthrough systems such as these may reveal previously unrecognized roles for H-NOX in *V. cholerae* pathogenesis and potential strategies for managing this and other potentially deadly pathogens through NO signaling pathways. In any case, with such a wide species distribution and diversity of functions, the H-NOX family will continue to shape what we know about bacterial signaling among pathogenic and non-pathogenic species alike.

## Abbreviations

DGC: diguanylate cyclase
EPS: extracellular polymeric substance
HK: histidine kinase
H-NOX: heme nitric oxide/oxygen binding protein
NO: nitric oxide
PDE: phosphodiesterase
Qrr: quorum regulatory RNA
RR: response regulator
sGC: soluble guanylate cyclase
